# A complex intervention to improve life experience during and after acute treatment for breast cancer: Preliminary results from intervention development for the Continuum PAROLE-Onco 360 program

**DOI:** 10.17269/s41997-025-01095-5

**Published:** 2026-01-13

**Authors:** Marie-Pascale Pomey, Hermann Nabi, Marie-Andrée Coté, Monica Iliescu Nelea, Asma Boubaker, Cécile Vialaron, Louise Normandin, Carole Lesperance-Huot

**Affiliations:** 1 Research Centre of the University of Montreal Hospital Centre, Montreal, QC Canada; 2 Chair in Evaluation of State-of-the-Art Technology and Methods, University of Montreal Hospital Centre, Montreal, QC Canada; 3 Centre of Excellence on Partnership with Patients and the Public, Montreal, QC Canada; 4 Department of Health Policy, Management and Evaluation, School of Public Health, University of Montreal, Montreal, QC Canada; 5 Department of Family Medicine and Emergency Medicine, Faculty of Medicine, University of Montreal, Montreal, QC Canada; 6 Research Centre of the University Hospital Centre of Québec, Université Laval, Quebec, QC Canada; 7 Cancer Research Center, Université Laval, Quebec, QC Canada; 8 Department of Social and Preventive Medicine, Faculty of Medicine, Université Laval, Quebec, QC Canada; 9 University of Montreal Hospital Centre, Montreal, QC Canada

**Keywords:** Breast cancer, Complex interventions, Patient co-investigators, Accompanying patient, Peer support, PAROLE-Onco 360 Continuum, Transition of care, Cancer du sein, Interventions complexes, Patientes co-chercheures, Patientes accompagnatrices, Soutien par les pairs, Continuum PAROLE-Onco 360, Transition de soins

## Abstract

**Introduction:**

In Quebec, 87% of women diagnosed with breast cancer report lingering after-effects, such as fatigue, chronic pain, and difficulty returning to work after treatment. The Continuum PAROLE-Onco (CPO) 360 program addresses these complex and various unmet needs through a comprehensive and patient partnership intervention.

**Objectives:**

The objectives of this study are to (1) explore the development process of the CPO 360 program during its inaugural year, with particular focus on the co-construction approach involving patient co-investigators (PCIs) and accompanying patients (APs), and (2) identify key insights to inform subsequent phases of feasibility and pilot testing, evaluation, and implementation, in alignment with a framework on complex interventions.

**Methodology:**

A qualitative multiple-case study was conducted across three Quebec university hospitals to evaluate the first year of the CPO 360 program’s implementation. The study focuses on how key components of this complex intervention were developed and adapted within real-world settings. Data sources included program documents, semi-structured interviews with accompanying patients, and a focus group with patient co-investigators. Thematic analysis was used to interpret the data, and the findings were reported in accordance with the COREQ checklist for qualitative research.

**Results:**

The findings highlight several key developments: the creation of training programs for accompanying patients (APs), the design of tools to support patients during care transitions, challenges related to data access and the implementation of needs-based stratification, the development of strategies to educate and engage healthcare professionals, and efforts to raise awareness among primary care teams regarding patient follow-up.

**Conclusion:**

This first phase in the development of the CPO 360 intervention is part of a dynamic, iterative, creative process that is open to change and forward-looking in its implementation. The co-construction process involving patients, professionals, and the community from the outset enables the development of a shared vision of how to improve the transition process in three different environments. Adjustments to its implementation will need to take account of intersectoral coordination and access to clinical resources.

## Résumé

**Introduction:**

Au Québec, 87% des femmes qui reçoivent un diagnostic de cancer du sein rapportent des séquelles persistantes, telles que la fatigue, des douleurs chroniques et des difficultés à retourner à la vie professionnelle après la période de traitements. Le programme Continuum PAROLE-Onco (CPO) 360 répond à ces besoins complexes et variés non satisfaits grâce à une intervention complète et un partenariat avec les patients.

**Objectifs:**

Les objectifs de cette étude sont les suivants: (1) explorer le processus de développement du programme CPO 360 au cours de sa première année, en mettant particulièrement l'accent sur l'approche de co-construction impliquant des patients co-chercheurs (PCI) et des patients accompagnateurs (AP), et (2) identifier les enseignements clés qui permettront d'orienter les phases suivantes de faisabilité et d'essais pilotes, d'évaluation et de mise en œuvre, conformément à un cadre sur les interventions complexes.

**Méthodologie:**

Une étude qualitative de cas multiples, menée dans trois hôpitaux universitaires du Québec, a évalué la première année de mise en œuvre du programme CPO 360, en se concentrant sur la façon dont les composantes clés de cette intervention complexe ont été développées et adaptées dans des contextes réels. Les sources de données comprenaient des documents en lien avec le développement du programme, des entrevues semi-structurées avec des patients accompagnateurs et un groupe de discussion avec des patients cochercheurs. Les données ont été analysées de manière thématique et l'étude a été rapportée en suivant la liste de contrôle du COREQ pour les rapports de recherche qualitative.

**Résultats:**

Les résultats montrent l’évolution de la création d’une formation pour les PA; les outils pour faciliter la transition des patientes; les limites d’accès aux données et de mise en œuvre de la stratification des besoins; le développement de stratégies de sensibilisation et formation des professionnels de la santé, et les manières de sensibiliser la première ligne au suivi de ces patientes.

**Conclusion:**

Cette première phase du développement de l'intervention CPO 360 s'inscrit dans un processus dynamique, itératif et créatif, ouvert au changement et tourné vers sa mise en œuvre. Le processus de co-construction impliquant dès le départ les patients, les professionnels et la communauté permet de développer une vision partagée de l'amélioration du processus de transition dans trois établissements différents. Les ajustements pour son implantation devront tenir compte de la coordination intersectorielle et de l'accès aux ressources cliniques.

## Introduction

Breast cancer is the most common cancer among women in Canada, accounting for approximately 25% of all cancer cases (Gouvernement du Canada, 2025). In 2024, there will be an estimated 30,500 new cases of breast cancer, with a 5-year survival rate of 89% due to advances in screening and treatment (Canadian Cancer Society, s. d.). In Quebec, 7000 women will be diagnosed with breast cancer, and more than 1000 will die of it (Gouvernement du Québec, 2025). Advances in the fight against cancer have improved breast cancer survival rates, although the rates vary according to the stage of the disease at the time of diagnosis. Five-year survival is estimated at almost 100% for women diagnosed at stage I, 92% at stage II, 74% at stage III, and 23% at stage IV (Société canadienne du cancer, 2024).

The post-treatment phase of breast cancer presents many challenges for patients. According to the Quebec Breast Cancer Foundation, a significant proportion of women report persistent after-effects such as chronic fatigue, pain, cognitive disorders, financial problems, emotional scarring, internal damages such as digestive/gastro-intestinal issues and difficulties reintegrating their personal life and work life, physical and reproductive limitations, DNA family issues, and dramatic anatomical changes (Fondation cancer du sein du Québec, 2025). These observations underscore the importance of an integrated care approach that considers not only clinical aspects, but also the psychosocial and personal needs of patients. Several models of survivorship care have been proposed to address these challenges, including risk-stratified follow-up, multidisciplinary care coordination, and integration of community resources (Horn et al., 2024; Mayer & Alfano, 2019; Urquhart et al., 2022). These approaches emphasise the importance of personalised support tailored to individual patient profiles and highlight the potential of structured peer support to help bridge existing gaps in care.

Post-treatment follow-up in oncology is focused mainly on clinical and biological parameters, often neglecting a holistic view of patients’ needs (Arsenault et al., 2024). Overuse of oncology services limits access to in-depth support, leaving many patients to cope with unanticipated side effects, an increased risk of recurrence, and psychosocial complications (Sciotto et al., 2017). This underscores the need for an integrated, seamless model of care that meets the diverse needs of patients who have completed the acute phase of their treatments.

The Continuum PAROLE-Onco (CPO) 360 program has been developed in response to these challenges and is being tested in the breast cancer trajectory. Its aim is to improve patients’ life experience by providing personalised support, from diagnosis and continuing throughout the transition to life after cancer, or post-acute treatment for those with metastatic cancer. The CPO 360 program is based on a multidimensional approach, integrating peer support (accompanying patients), personalised learning tools, and coordinated follow-up among oncology, primary care, and community services according to the patient’s needs. The program favours co-construction with patient co-investigators (PCIs) and accompanying patients (APs), actively involving them as key players in the design and development of the interventions in CPO 360.

CPO 360 consists of several interventions, making it a complex program due to its multidimensional components and the need to coordinate the various players to ensure consistency and effectiveness (Skivington et al., 2021). These interventions are (1) the development and use of various teaching strategies for patients in support of their transition; (2) accompaniment provided by an accompanying patient throughout the trajectory; (3) a personalised learning plan tailored to the patient’s needs, accessible on a digital platform; (4) stratification of patient needs according to various components, enabling patients to be directed toward the structures best suited to their situation; (5) awareness-raising and training of cancer professionals in the new care model; and (6) awareness-raising and training of primary care professionals and involvement of community services. Since September 2023, this program has been funded by the Canadian Institutes of Health Research (MP-02–2024–11860) for a 4-year period and is currently being implemented in three facilities in the province of Quebec, Canada.

These various components draw on several approaches to meet the varied needs of patients, as they cannot be fully met by a single intervention, require the collaboration of multiple players from different sectors, and adopt strategies adapted to a diverse range of patient profiles (Horn et al., 2024).

In the context of evaluating complex interventions, the UK Medical Research Council (MRC) framework proposes four phases: development, feasibility/pilot test, evaluation, and implementation (Craig et al., 2008). This article focuses on the development phase, which is grounded in a co-construction process. Specifically, it examines the period between the initial emergence of an intervention idea and its formal pilot testing in the subsequent phase (Bartholomew Eldredge et al., 2016); Bleijenberg et al., 2018; Hoddinott, 2015; O’Cathain et al., 2019a, 2019b).

The objectives of this study are: (1) to examine the intervention development process for the CPO 360 program during its first year, with a focus on the co-construction process involving patient co-investigators (PCIs) and accompanying patients (APs); and (2) to identify key insights to inform subsequent phases of feasibility/pilot test, evaluation, and implementation which takes up the UK Medical Research Council (MRC) framework.

## UK Medical Research Council (MRC) framework

The MRC framework (O’Cathain et al., 2019a, 2019b) proposes 11 actions in the first phase of development. As recommended by the authors, it may not be possible or desirable for developers to address all these actions during their development process, and indeed some may not be relevant to every problem or context. For our study, we therefore selected the following actions: (1) plan the development process; (2) involve stakeholders, including those who will deliver, use, and benefit from the intervention; (3) assemble a team and establish decision-making processes; (4) draw on existing theories; (5) articulate the theoretical basis for the program; (6) undertake primary data collection; and (7) understand the context (see Table 1). We did not retain the following actions: review published research evidence; pay attention to future implementation of the intervention in the real world; design and refine the intervention; and end the development phase. The reasons are that we plan to publish reviews on the various themes covered by our complex intervention and, second, as part of the intervention pilot process, we will be paying attention to program implementation in the real world and will continue to collect preliminary data to enable us to refine the program.

**Table 1:** Framework of actions for intervention development (O’Cathain A, Croot L, Duncan E, et al. Guidance on how to develop complex interventions to improve health and healthcare. *BMJ Open* 2019;9:e029954. doi:10.1136/bmjopen-2019–029954)

Action	Dimensions covered
1. Plan the development process	Identify the problem to be targeted and refine understanding of it throughout the process Assess whether the problem is a priority Consider which aspects of the problem are amenable to change Ask whether a new intervention is really needed and if the potential benefit of the new intervention justifies the cost of development. Determine the time needed to undertake intervention development Obtain sufficient resources/funding for the intervention development study Draw on one or more of the many published intervention development approaches, recognising that there is no evidence about which approach is best and apply flexibly depending on the problem and context. Involve stakeholders during the planning process (see next Action) Produce a protocol detailing the processes to be undertaken to develop the intervention
2. Bring together a team and establish decision- making processes	Include within the development team individuals with relevant expertise: in the problem to be addressed by the intervention including those with personal experience of the problem, in behaviour change when the intervention aims to change behaviour, in maximising engagement of stakeholders and with a strong track record in designing complex interventions. It may be hard to make final decisions about the content, format and delivery of the intervention, so only some team members may do this. There is no consensus about the size or constituency of the team that makes these final decisions, but it is important early on to agree a process for making decisions within the team
3. Involve stakeholders, including those who will deliver, use and benefit from the intervention and those integrated on the research side (especially patient co-investigators)	Work closely with relevant stakeholders throughout the development process: patients, the public, the target population, service providers, those who pay for health and social services or interventions, policymakers, and intervention design specialists Develop a plan at the start of the process to integrate public and patient involvement into the intervention development process Identify the best ways of working with each type of stakeholder, from consultation through to coproduction, acknowledging that different ways may be relevant for different stakeholders at different times Use creative activities within team meetings to work with stakeholders to understand the problem and generate ideas for the intervention Ensure the development of the research component with the participation of patient co-researchers
4. Draw on existing theories	Identify an existing theory or framework of theories to inform the intervention at the start of the process, for example, behaviour change or implementation theory Where relevant, draw on more than one existing theory or framework of theories for example, both psychological and organisational theories
5. Articulate program theory	Develop a program theory. The program theory may draw on existing theories. Aspects of the program theory can be represented by a logic model or set of models Test and refine the program theory throughout the development process
6. Undertake primary data collection	Use a wide range of research methods throughout, for example, qualitative research to understand the context in which the intervention will operate, quantitative methods to measure change in intermediate outcomes
7. Understand context	Understand the context in which the intervention will be implemented. Context may include population and individuals; physical location or geographical setting; social, economic, cultural, and political influences and factors affecting implementation, for example, organisation, funding, and policy

## Methodology

### Design

A qualitative multiple-case study was conducted to evaluate the first year of development of the CPO 360 program in three university hospitals in the Province of Quebec, Canada, with a focus on 7 out of 11 MRC actions (Creswell & Creswell, 2013).

### Data sources

Two sources of data were used: documents relating to the activities carried out during this first year, and interviews and focus groups conducted with the stakeholders involved in this phase, including managers, health professionals, researchers, PCIs, and APs.

### Data collection and analysis

All meeting minutes from September 2023 to October 2024, along with recordings from the two collaborative workdays involving all CPO 360 participants, were analysed to shed light on how the various projects operated and how stakeholders collaborated.

In addition, nine (*n* = 9) interviews were conducted with APs (Facility 1, *n* = 3; Facility 2, *n* = 4; and Facility 3, *n* = 2) and a focus group was held with the three PCIs between August and October 2024. Another focus group was organised with members of the governance committee (*n* = 12), which includes managers, medical managers, researchers, PCIs, and APs. The purpose of the interviews with the PCIs and APs and of the focus group with the governance committee members was to gather data on the roles they played in the development of the various projects, the obstacles they encountered, and the lessons learned, in preparation for the CPO 360 development. The second focus group was used to evaluate the role played by the PCIs in the research and in coordinating the various projects. The interview and focus group guides are provided in Appendix 1. The interviews and focus group discussions were recorded and transcribed. The transcripts were coded and analysed using NVivo software (Wanlin, 2007), applying the thematic analysis method (Hervé Stecq, 2014) to ensure rigour and transparency in the interpretation of results. The study followed Consolidated Criteria for Reporting Qualitative Research (COREQ) guidelines to ensure high-quality and reliable results (Gedda, 2015).

## Results

We present the results according to the seven dimensions of the MRC framework.

### Plan the development process

The idea for this project arose from several situations that created a window of opportunity to propose CPO 360. First, some members of the research team had previously worked on two oncology projects: the PAROLE-Onco program (Pomey et al., 2021) and the Bridge to Home project (Arsenault et al., 2024). These projects highlighted the need for peer support after the end of treatment, and for tools to help patients and teams better cope with the various issues associated with the post-treatment period. In addition, the oncology teams highlighted the difficulties inherent in monitoring both on-treatment and post-treatment patients simultaneously. These observations echoed those found in the literature (Forsythe et al., 2014; Hobden et al., 2021). These two projects have therefore contributed to building valuable expertise in integrating accompanying patients (APs) into the breast cancer care pathway. They have also led to the development of a practical toolbox designed to enhance collaboration between oncology specialists and primary care professionals (Chaire de recherche en évaluation des technologies et des pratiques de pointe du CHUM-Engagement des citoyens et des patients dans la transformation des organisations et du système de santé,^2023^) and to support patients in gaining a better understanding of their disease and the impact of treatment on their quality of life. In addition, the Quebec Ministry of Health and Social Services in Canada has identified improving the transition between cancer care and follow-up to prevent recurrence as a priority (Ministère de la Santé et des Services sociaux (MSSS), 2024) and to use the toolbox developed in one facility as part of the Bridge to Home project for all the other facilities in the network (Chaire de recherche en évaluation des technologies et des pratiques de pointe du CHUM-Engagement des citoyens et des patients dans la transformation des organisations et du système de santé, 2023). Lastly, another part of the research team is specialised in breast cancer and the epidemiological stratification of this patient group in terms of care and service needs (Urquhart et al., 2022).

*Bridge to Home has enabled us to work with patients and professionals on a toolbox for both professionals and patients. *(Researcher)

*I was lucky enough to work on the development and implementation of PAROLE-Onco as a patient co-researcher, and I was also able to help evaluate the program’s effects. One of the areas for improvement was not to end the accompaniment when people finish their curative treatments. The post-curative treatment period is critical, and patients find themselves grappling with situations they hadn’t anticipated. *(Patient co-researcher)

It was in this context that the CPO 360 project was proposed to CIHR and funded for 4 years (Gouvernement du Canada, 2015), in order to co-construct the program with the three establishments that had implemented PAROLE-Onco in the breast cancer trajectory, allowing flexibility in the way it was implemented according to the specific contexts of these facilities (see Table 2 for the list of facilities). A protocol has been submitted to the ethics committee of the three facilities and is currently being published (Pomey et al., 2025).

**Table 2:** Characteristics of the three facilities

	**Facility 1 (F1)**	**Facility 2 (F2)**	**Facility 3 (F3)**
Location	City of Quebec area	Montreal area	Montreal area
Type of facility	University hospital	Integrated university health and social services centre	University hospital
Number of employees	14,000 +	17,500 +	14,000 +
Number of physicians	1700 +	947 + (327 general practitioners and 620 specialists)	1000 +
External partners	University, affiliated research centres	Universities, community organisations	University, research institutes
History of AP integration	Genetic predisposition for breast cancer	Multiple cancer trajectories	The entire breast cancer trajectory
Educational material	Oncology reference and information service, dedicated centre for people with a genetic predisposition, workshops, conferences, and training courses	Patient space, association, and information centre for patients and their families	Health fact sheets available to patients on the facility’s website In-house patient association
Collaboration with primary care for patient follow-up	No	No	No

### Involve stakeholders including those who will deliver, use, and benefit from the intervention

As CPO 360 will be implemented in the same facilities as those that have implemented PAROLE-Onco (see Table 2), the research team was already familiar with the relevant stakeholders involved in oncology and, more specifically, in the breast cancer trajectory. In addition, APs had already been recruited, trained, and integrated into the teams, and were thus immediately involved in the development of the intervention.

From the outset, the research team was composed of three co-principal investigators, including one patient co-principal investigator—and three additional patient co-investigators, one representing each site. For several years, the team has systematically involved patients in every stage of its interventional research projects. (Pomey et al., 2021; Pomey et al., 2025; Potvin et al., 2021).

Since the facilities have detailed knowledge of the environments with which the intervention would be developed, working mechanisms were discussed with each of them to determine which stakeholders should be informed, consulted, and brought in to participate in the co-construction of the program.

A project governance committee was quickly set up, comprising three representatives from each facility: a manager, a medical manager, and an AP, as well as the three co-principal investigators (one qualitative researcher, one quantitative researcher, one PCI). This governance committee held brainstorming meetings to work on the various components of CPO 360 and better define the environment in which the program would be implemented.

*We already know the research team and are used to working with them. We have therefore quickly established the working arrangements we wish to put in place. *(Researcher)

*I’m already an AP within PAROLE-Onco, it makes sense to continue working on Continuum PAROLE-Onco. I’m on one of the committees to ensure that CPO will be well integrated into the APs’ activities.* (PCI)

A research committee was also set up, with parity between career researchers (*n* = 4) and co-investigators (*n* = 4), and incorporating the best practices in patient-oriented research proposed by CIHR (Canada’s Strategy for Patient-Oriented Research, 2011) and Eupati (Education That Empowers– EUPATI, 2025).

In addition, two 1-day meetings were held in September 2023 and 2024 to review progress on project development and discuss objectives for the coming year with all the stakeholders (Journée annuelle projet Continuum-PAROLE-Onco, 2024; Journée lancement projet Continuum-PAROLE-Onco, 2023).

### Bring together a team and establish decision-making processes

To develop CPO 360, five projects have been set up: (1) creation of a tool box to support patients throughout their journey, including a personalised learning path (PLP) (Guise et al., 2017) including a chatbot (Chen et al., 2025; Lawson McLean & Hristidis, 2025; Ma et al., 2024; Xu et al., 2021); (2) development of a specific training module for APs to help them better understand the new activities entrusted to them; (3) development of algorithms to help clinicians provide personalised follow-up care to patients, based on their clinical data and specific needs; (4) development of tools and training for healthcare professionals working in oncology departments; (5) development of tools and training for primary care professionals and mobilisation of community services. A summary of activities is presented in Table 3, which includes the APs’ and PCIs’ perceptions of their involvement in these activities, as well as the leadership provided by each facility.

**Table 3:** CPO 360 program projects

Projects	Objectives	Working methods	PCIs’, APs’, and governance committee members’ perceptions	Rôles des établissements
1. Creation of a tool box to support patients throughout their journey, including a personalised learning path (PLP) including a chatbot	Identify the tools developed in the Bridge to Home project and those proposed by the Ministry of Health and Social Services, and create a toolbox adapted to each facility	The project governance committee has identified the tools proposed by the Ministry of Health and Social Services Bridge to Home and each facility. Tools were pre-tested with target users during workshops and adapted based on feedback. The tools included in this kit cover a tool to assess needs at the end of treatment and self-care capacity, a stratification tool to define follow-up methods. All these tools have been further developed by APs at one of the three facilities to ensure that they meet patients’ needs	“*I was part of a working group with 3 other patients. We looked at all the tools proposed by the Ministry and tried to simplify them. One of the tools—the oncology health passport—enables patients to record all this information in one place. My facility will start using it in the next year.*” (AP)	All three facilities are mobilised at the same level
	Creation of a personalised learning path including a Chatbot that enables patients to find, at any time and in one location, information about their journey with the disease and information to help them develop self-management skills	A working group composed of patient partners, clinicians, and managers worked on an adaptation of the PLP oncology to a specific on the breast cancer pathway including questions that can be included in a chatbot. Each component was tested iteratively through working groups. Medical experts (*n* = 6) were also consulted to validate the information sources and supplement them	“*We were invited to work with the PLP, which was developed with other patients than us in a general way. I’m looking forward to working with it more directly*.” (AP) “*I helped develop the interview grids for the chatbot and I was also able to take part in one of the focus groups. I was very impressed with the quality of the discussions and how similar our observations were*.” (PCC)	F3 has a health literacy department and has already had experience of developing a PAP. It has therefore taken the lead in developing the PAP
2. Development of a specific training module for PAs to help them better understand the new activities entrusted to them	Training module for APs to enable them to familiarise themselves with the toolbox, gain a better grasp of transition-related issues and intervene on the front line	Création d’un groupe de travail avec des PA avec le soutien de professionnels spécialisés en andragogie	“*I’m involved in the whole process of creating training programs through the Faculty of Continuing Education. I really appreciate their openness and ability to consider all my suggestions.*” (PCI) “*For several years now, I’ve been providing training in my facility, and I love this direct contact with patients. Now I’m contributing to online training and working to ensure that these two levels of training complement each other.*” (AP) “*These new modules are really highly relevant to us being better equipped to respond to more complex issues.*” (AP)	F1 and 3 have specific resources for developing pedagogical content and have participated in developing the content of the first training course offered to APs. They are therefore more involved in the form and content of this module
3. Development of algorithms to help clinicians provide personalised follow-up care to patients, based on their clinical data and specific needs	Develop algorithms to help clinicians provide personalised follow-up care to patients, based on their clinical data and specific needs	The three facilities were asked to share patient follow-up data from the last 10 years. Facility 1 (F1) is the only one to have built up a bank of easily accessible data over the last 20 years. Through its dedicated medical data centre, F3 was able to extract 10 years of clinical data on breast cancer patients from its information systems. Facility 2 (F2) only has data collected by trained registrars, whose mission is to enter data in the Quebec cancer registry Analysis of the data extracted from the three databases can now begin, under the responsibility of one of the co-principal investigators (HN)	“*This part is difficult. The data access rules are complex, and for two of the three sites, we’re still waiting to be able to use them. As a patient, it isn’t easy to follow and understand all the nuts and bolts of it.*” (PCI) “*During this first year, we came up against many constraints. The investigators were able to do some analysis, but they weren’t able to involve the patient co-investigators in the process and analysis as much as initially planned.*” (PCI)	F1 is the only one to have built up a bank of easily accessible data over the last 20 years. Through its dedicated medical data centre, F3 was able to extract 10 years of clinical data on breast cancer patients from its information systems. Algorithms will be tested at those facilities
4. Development of tools and training for healthcare professionals working in oncology departments	Develop training courses to raise awareness and train healthcare professionals working in oncology departments of the post-treatment transition and in the use of the tools developed in the CPO 360 program	A workshop facilitated by the three co-principal investigators (including the patient co-PI) was held with all the breast cancer trajectory stakeholders in each site. Among the avenues explored, emphasis was placed on standardising transition practices, improving communication among the stakeholders, and developing educational tools to support patients in their self-management (see Project 1). This discussion has also paved the way to construct a course with a subgroup of clinicians	“*We started a working group at our facility. I was able to see professionals involved in getting things moving. They weren’t familiar with the Ministry’s tools, and I was able to present the tools to them despite their limitations, but it’s important to get the word out about CPO 360.*” (AP) “*It isn’t easy to get into the field with clinicians and managers. For now, I haven’t found my place in the discussions. But I do bring up situations that concern me and that I have to face as an AP*.” (AP) “*It isn’t easy for accompanying patients to find their place here, but at the same time, it’s thanks to our personal accounts that the professionals will get moving!*” (PCI) “*I think we’ve developed high-quality training for the APs. Now we need to tackle training for the professionals and managers. They’re still having a hard time understanding how to integrate us into their teams.*” (AP)	All three facilities are mobilised at the same level
5. Development of tools and training for primary care professionals and mobilisation of community services	Develop workshops for primary care teams and community services on CPO program	An inventory of community resources to support the transition has been realised A questionnaire has been developed to survey the primary care settings, based on the literature and drawing on previous work A scoping review is being undertaken to analyse the different models in place in order to mobilise primary care in the follow-up of cancer survivors	“*Everything needs to be done. It’s a big project. I don’t know if we’ll have what it takes to complete it.*” (AP) “*I’m looking forward to serving as a link between the hospital and primary care settings. I could see myself working closely with family medicine groups and community organizations.*” (AP)	All three facilities are mobilised at the same level

### Draw on existing theories and develop program theory

**Fig. 1:**
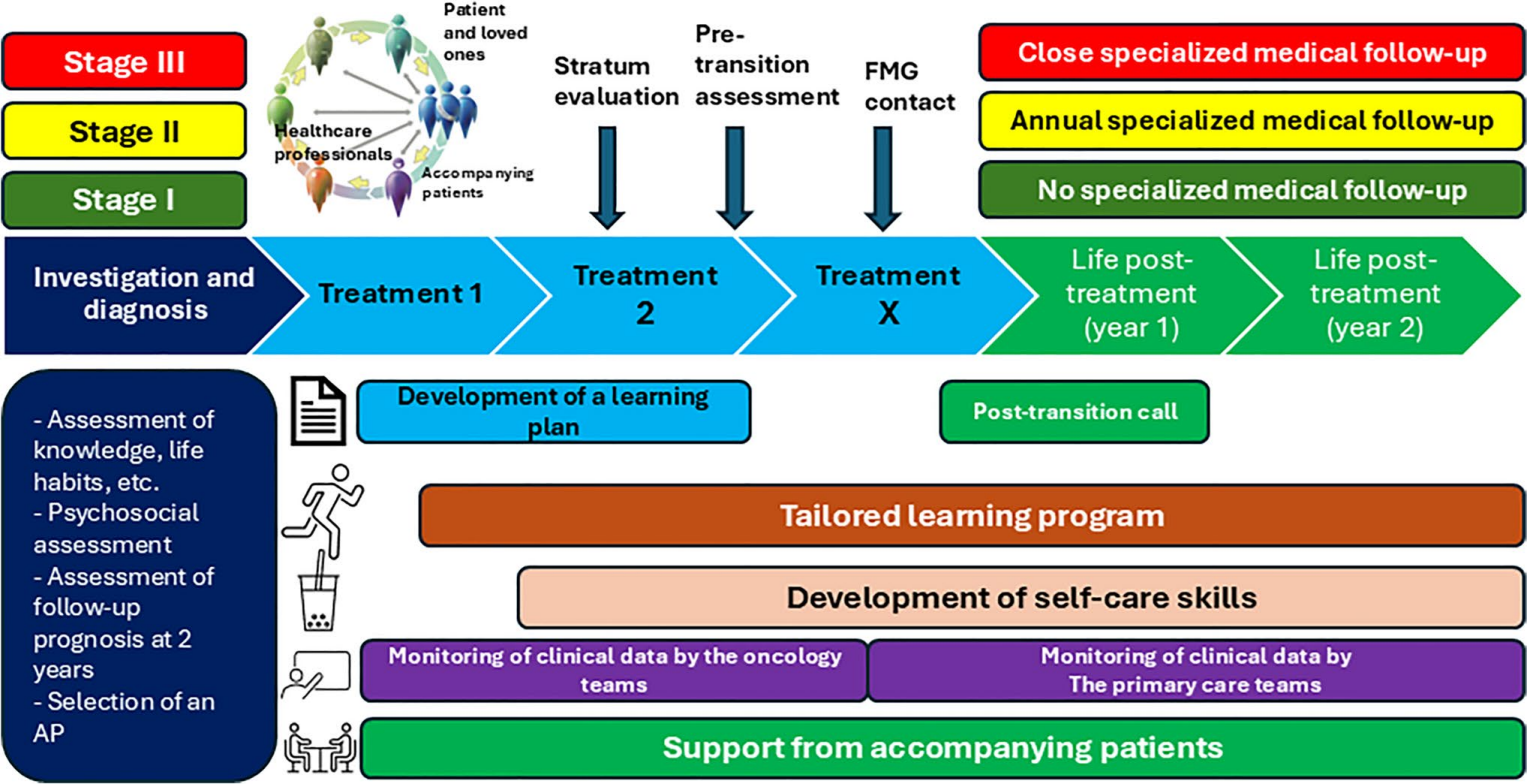
CPO 360 model diagram

To set up CPO 360, we built a theoretical framework based on Donabedian’s framework (Donabedian, 1966), taking into account resources, processes, and outputs/outcomes starting with the resources to be mobilised, the different workstreams implemented, and the results based on the quintuple AIM (Nundy et al., 2022), which is recognised in the province of Quebec as the reference framework for measuring the effects of interventions. This framework was co-constructed by the project governance committee (see Fig. 1).

**Fig. 2:**
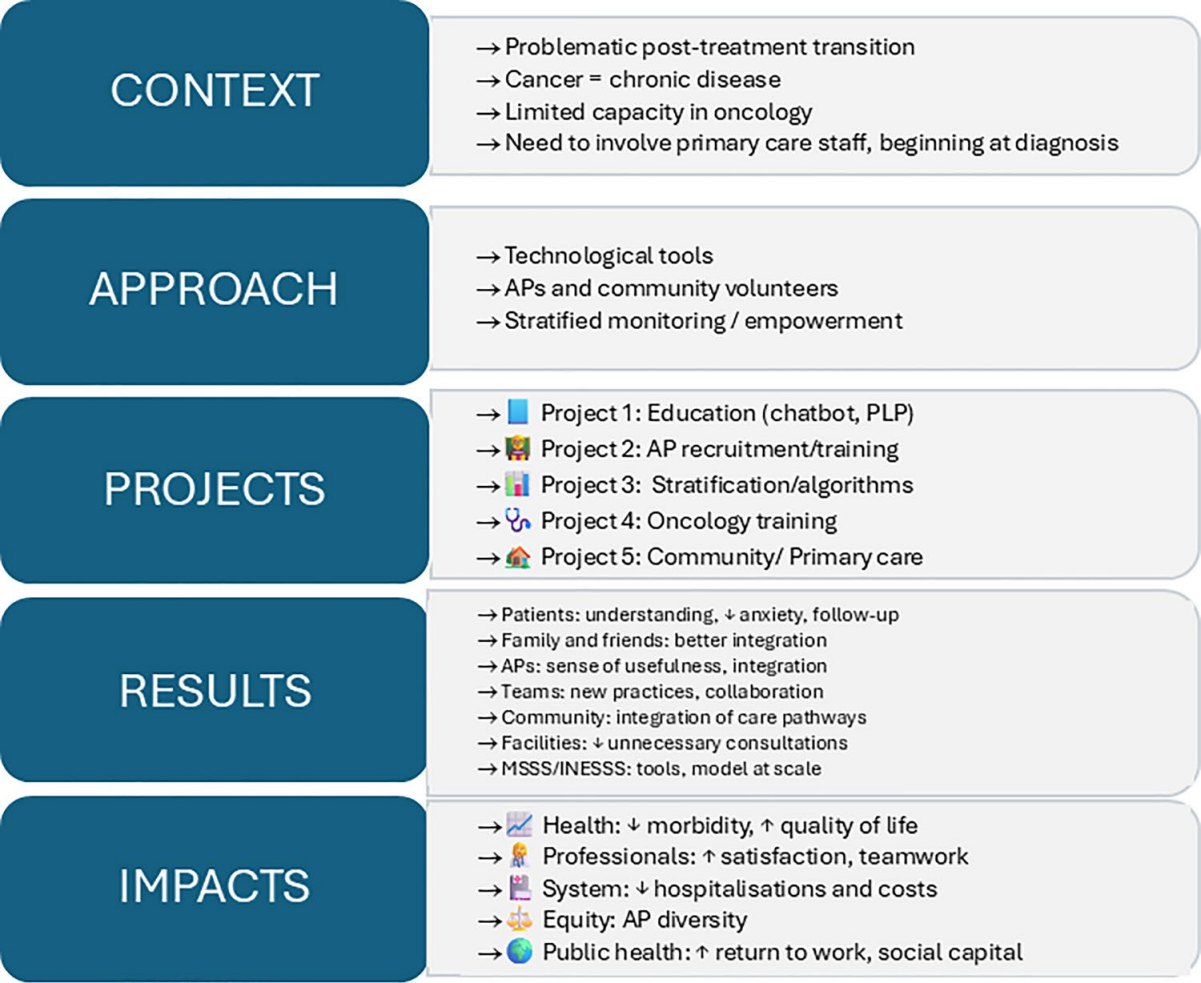
CPO 360 logic model

Based on this framework, the project governance committee also produced a logic model, detailing the program theory and specifying how the various workstreams will act to achieve the objectives (see Fig. 2 and for the original version of the logic model see Appendix 2).

In addition, the team identified the Framework for Developing and Evaluating Complex Interventions (Skivington et al., 2021) as the basis for its theory of actions to implement strategies that will support the management of psychological and organisational change (see Table 1).

### Undertake preliminary data collection

Data were collected from APs, PCIs, and members of the governance committee, and all documents produced throughout this development phase of the intervention were tracked. This data collection enabled us to gain a better understanding of how these actors had appreciated certain initiatives and highlighted the difficulties they encountered. These stakeholders’ perceptions of each project are presented in Table 3.

In terms of the PCIs’ perception of how the project was run, they particularly appreciated the way the team worked together:

*I attend all the meetings. I like this collaboration, where we’re involved in all the decisions and we’re not considered just tokens, but rather full members of the research team. (…) Among the examples of interventions I’ve made, I’ve brought…*(PCI)

*For the first time in my life, I participated in the development of logic models, and I really enjoyed the exercise. I learned a lot about how to better understand the various interventions and how they can interact with each other.* (PCI)

This was also the case for the other members of the governance committee:

*Patient co-researchers are very involved in the project. They are present at all the meetings. In fact, they often attend more than we do... they come up with relevant ideas that help us avoid getting lost in the details and administrative complexities. Their presence allows us to move forward, focus on our objectives and, above all, on the well-being of our patients.* (Manager)

*It’s the first time I’ve worked with patient co-researchers, and despite some initial misgivings, I really appreciate their presence. They’re very committed, ask good questions that force us to go further, and are a source of many pertinent ideas that we can translate into concrete action.* (Medical manager)

On a more negative note, the PCIs raised their difficulties in understanding the complexity of the administrative procedures, which also echoes the feedback received from the other members of the steering committee:

*I really enjoy working on research tools, even though I sometimes find the process long and don’t understand why it takes so much time to sign contracts between facilities, and to receive the ethics certificate.* (PCI)

*It wasn’t easy to obtain ethics certificates for all three establishments and to set up collaboration agreements to exchange data. This led us to fall behind schedule.* (Manager)

### Understand context

The three institutions participating in CPO 360 are facilities with which the researchers have been working since 2017 (Pomey et al., 2021). However, they have undergone personnel changes in terms of cancer program governance. Therefore, for each facility, a working group was set up, modelled on a continuous improvement committee (Pomey et al., 2018). Its first mission was to take stock of the situation regarding the involvement of APs in the breast cancer trajectory following the implementation of PAROLE-Onco in order to consolidate its achievements and hold a meeting with all the people involved in the trajectory as mentioned in section “Involve stakeholders including those who will deliver, use, and benefit from the intervention.”

## Discussion

These preliminary results confirm that this program is what is called a “complex intervention,” as it includes many components, aims to change the behaviour of various populations, requires the expertise and skills of the people providing and receiving the intervention, mobilises various groups of people, and must be flexible in the implementation of these different components depending on the setting (Ansell & Gash, 2008; Blencowe et al., 2019).

Using the MRC framework, we were able to take into account the different facets of developing this complex intervention, notably the importance of adaptation to context, stakeholder involvement, and iterative refinement. In particular, Projects 1 and 2 illustrate how structured tools and training can be co-developed to support patient self-management and continuity of care. Moreover, the integration of peer support and stratified follow-up approaches is consistent with emerging models in survivorship care (Alfano et al., 2022; Urquhart et al., 2022). The Personalized Learning Plan (PLP) and Chatbot, while still in their exploratory stages, are being developed through co-construction with patients and professionals, reflecting a participatory, responsive design process that aligns with these evolving care models.

Regarding the interactions between the intervention and its context, the five projects focus on the characteristics of the three institutions involved highlighting their strengths and their interest in participating in these projects. For example, F3 provided expertise on building the training program and the PLPs. F2 took the lead in making the toolkit provided by the Ministry accessible to all patients. And F1 is serving as the leader of Project 3, processing the administrative data and developing algorithms to support decision-making. These initial results show that, in an entirely unplanned way, the institutions have developed leadership in different areas, allowing each to find its place while complementing the others’ contributions.

However, for Project 5, which is focused on oncology teams, primary care and the community environment, taking the initiative appears to be a more complex task for the institutions. In the coming years, it will be interesting to see how these projects will be handled.

As for the program’s theory, the researchers worked on developing the logic model for CPO 360 and have allowed the PCIs to be stakeholders in the process. This logic model will need to be reviewed annually to ensure that the work carried out in the projects supports the expected effects.

Through the active participation of the various actors, the various facets of the project have been co-constructed during this first year. Based on feedback from the APs and PCIs, it was possible to better understand the contribution made by patients in each of the projects. A more formal evaluation framework for their participation throughout the project will be implemented to ensure that we capture how they influence the various stages and projects (Centre d’excellence sur le partenariat avec les patients et le public (CEPPP), 2023). Such work will continue to enrich the literature on the implementation of complex interventions and the participation of patients in their construction and evaluation (Contandriopoulos et al., 2011).

Regarding the uncertainties around running the project, various findings emerge from these initial results. We will discuss them by suggesting avenues for improvement to further develop the project and ensure its optimal implementation over the next 3 years.

Regarding the difficulties in obtaining an ethics certificate for a multi-centre study within a reasonable time frame, the rules for multi-centre studies (Contandriopoulos et al., 2011) did not allow data collection until 7 months after the project was submitted. This will make it difficult to complete the project within the deadlines imposed by the funding organisations. A second observation concerns the varying degrees of progress in the projects, which require considerable involvement of stakeholders from the three institutions who do not have the same degree of maturity and interest in participating. Projects 1, 2, and 4 are the most advanced, with each benefitting from shared leadership by the three institutions. For Project 4 on mobilising oncology service professionals, workshops will also be planned for the other two facilities (F1 and F2). For Project 3 on stratification, the data varied in quality, depending on the context. They highlighted difficulties encountered in mobilising the three facilities around a consistent development of algorithms. However, for Project 3, the quality of the data from F1 and the data provided by F3 should make it possible to propose algorithms that can then be tested in the third environment (F2). With regard to Project 5, focused on primary care, the integration of primary care clinical researchers into the research team should help create these bridges. In addition, patients’ involvement in the projects varied, depending on the project, with greater involvement in the projects that are more focused on the relationship (Cleemput et al., 2019; SPOR Evidence Alliance, 2020).

This first year also highlighted the importance of having an agile model in order to be flexible and open to the adjustments required to implement the project. The uncertainties that were identified, such as the time taken to obtain ethics certificates, highlight the importance of adopting an adaptive approach. By taking into account the uncertainties and specific features of each environment, CPO 360 will be able to consider the evolving needs of patients, healthcare professionals, and institutions.

### Limitations of the study

Although this study has provided significant preliminary results on the implementation of CPO 360 during this first year of implementation, some limitations should be noted.

First, the delays in obtaining the ethics certificate prevented us from conducting the interviews that had been planned with health professionals and managers at the three institutions. This limited our ability to have a diversity of perspectives in these preliminary results. One way these limitations were addressed was by seeking these points of view in the documents analysed.

## Conclusion

The CPO 360 program represents a significant advance in the care of breast cancer patients. By carrying out a complex intervention and emphasising the role played by PCIs and APs, this evaluation contributes to a better understanding of the various strategies employed to implement and evaluate it. These preliminary results show that the experience of the PCIs and APs in the co-construction of CPO 360 has allowed them to intervene in various areas that can be enlightened through their particular expertise. By continuing to evaluate this implementation over the next 3 years, CPO 360 will help develop a better understanding of the science of implementing complex interventions.

## Data Availability

All authors had full access to the data and materials. The data is available from the authors upon reasonable request.

## Appendix 1

**General questions**

**1. Motivations and the initial role**

○ What motivated you to become involved in the CPO 360 program as an accompanying patient or co-investigator?

○ How would you define your role in this program, and what do you think makes it unique?

**Project-specific questions**

**2. Project 1: Development of transition tools**

○ Did you help develop tools, such as the personalized learning path (PLP) or the chatbot? If so, what value did you add to their development? Which aspects were the most rewarding or the most difficult?

○ In your opinion, which tools best meet the needs of patients in their post-treatment transition?

**3. Project 2: Training for accompanying patients**

○ How were you involved in the development of the training program?

○ What did you think of the training modules that were developed or that are currently in development?

○ What aspects of the training should be strengthened to better equip accompanying patients for the role?

**4. Project 3: Stratification of needs and follow-up algorithms**

○ How do you perceive your involvement in the development of algorithms for patient stratification?

○ Did you take part in the workshop with the Facility 3 team?

○ Do you have any suggestions on ways to better integrate your views into this technical process?

**5. Project 4: Training of oncology professionals**

○ Did you collaborate with clinicians or managers on transition or training tools? If so, how did it go?

○ What do you see as the major challenges in raising awareness among professionals about fully integrating APs into their teams?

**6. Project 5: Mobilizing primary care and community services**

○ Do you see opportunities to improve the links between hospital services, primary care and community organizations?

○ What role would you like to play in raising awareness or in training primary care teams?

**7. Project 6: Research and collaborative governance (for patient co-investigators only)**

○ What has been your experience as a member of research and implementation committees?

○ What changes or adjustments would you suggest to strengthen your role in and contributions to project governance?

**Concluding questions**

**8. Lessons learned and points of view**

○ What lessons have you learned so far from your experience in the projects?

○ In your opinion, what are the most promising results or the most significant challenges ahead for CPO 360?

**9. General recommendations**

○ What would you recommend to enhance the program’s impact and improve the integration of APs and PCIs in future phases?

## Appendix 2

**Table:**

Context	Patient follow-up across the continuum of oncology care, from diagnosis to a return to life “after acute treatment for cancer,” represents a major public health issue. More and more patients are living with cancer as a chronic condition, which requires a follow-that is tailored to the context and the risk of recurrence. However, oncology departments do not have the capacity to monitor all new and chronic cancer patients with the same level of involvement over time. This project hypothesizes that the transition to life after acute treatment for cancer must be prepared, starting at the time of diagnosis, and that it is essential for patients not only to be partners in their own care, but also to be empowered, according to their risk and needs, to develop the self-care and adaptation skills that will enable them to attend to their own needs for specialized post-treatment care, alongside their primary care team and community support.											
Purpose	To improve quality of life throughout the continuum of oncology care, from diagnosis to return to the community											
Nature of the intervention	To implement technological tools (connected objects, a mobile application and a web portal) and deploy the time and expertise of patient partners and the time and knowledge of volunteer organizations											
Components of the intervention	Project 1: Patient teaching strategies		Project 2: Recruitment and training of accompanying patients	Project 3: Stratification of needs and follow-up algorithms			Project 4: Tools and training for healthcare professionals in oncology departments		Project 5: Awareness-raising and training for primary care professionals and community services			
	Patient learning path with a chatbot	360 toolbox	Development of expertise in AP recruitment	Development of new training modules	Collection of historical data	Development of algorithms	Development of tools for the transition	Development of training in the new transition model for professionals	Development of training	Synthesis of knowledge on models in primary care	Collaboration with community organizations	COMPAS+-type workshop
Inputs	CEPPP research team - APs	CIUSSSEMtl-MSSS research team	CEPPP-CHUM- ETMP Chair	Faculty of Continuing Education, University of Montreal- CEPPP-CHUM- ETMP Chair -APs	CHUQ-UL research team	CHUQ-UL research team	CHUQ-CHUM-CIUSSSEMTL research team	Continuing Education Faculty, University of Montreal - Research team	Continuing Education Faculty, University of Montreal - Research team	Work with residents in general medicine	ROCCO and other organizations	MSSS-INESSS
Activities	Creation of a working group to develop the PLP and the chatbot	Adaptation of the 360 toolbox	AP implementation guide (PAROLE-Onco) – done	Creation of a working group to develop these next modules	Analysis of historical cohorts	Cross-referencing of historical cohorts with algorithms used in the literature, and proposal of suitable algorithms	Creation of a working group to develop the kit for professionals	Creation of a working group to develop the training	Creation of a working group to develop the training	Knowledge synthesis	Reconciliation of activities carried out by APs and those by community organizations	Testing of a COMPAS+ workshop
Effects	Patients able to develop self-care skills, better anticipate the transition stages and use resources		Facility able to identify APs	APs empowered to develop patients' self-care skills and informational, educational, emotional and navigational support	Know the patient trajectories	Better understand the need for algorithms in clinical settings	Empower oncology professionals to use the kit and the new care model		Empower primary care professionals to use the kit and the new care model	Knowledge of models and publication of an article	Better collaboration with community groups	Closer ties between oncology and primary care
Results	FOR PATIENTS •Better understanding of the disease •Improved capacity for self-management •Reduced anxiety •Greater commitment to care •Change in life habits •Better use of resources •Reduced treatment side effects •Better follow-up in primary care •Better knowledge of community organizations and their contributions	FOR FAMILY CARE-GIVERS •Better integration in the team •Better knowledge of the disease and its effects •Better management of time and financial resources •Reduced anxiety •Ability to better support the person with cancer	FOR APs •Feeling useful/sharing one’s experiences •Mobilization of the AP community •Satisfaction with using one’s experiential knowledge •Better knowledge of the disease, its trajectories and medical/community resources •Better integration into the clinical team •Development of new transition-related skills	FOR CLINICAL ONCOLOGY TEAMS •Learn to work and enjoy working with APs in the clinic •Better integration of the transition’s complexity for patients •Better understanding of the role played by primary care in patient follow-up •Improved clinical and organizational practices •Better response to patients' needs and expectations •Improved collaboration by oncology and primary care teams •Support for patients making informed decisions and being partners in their care •Improved work satisfaction		FOR MANAGERS •Learning to work with and value APs' work at the clinic and organization •Knowing the program and spreading the word •Improving the quality of care and services •Learning to manage the resources required to implement the program •Learning how to mobilize clinical teams •Improved work satisfaction	FOR CLINICAL TEAMS IN PRIMARY CARE •Better integration into the patient care pathway •Better collaboration with oncology departments •Greater role in patient follow-up •Improved work satisfaction	FOR COMMUNITY ORGANIZATIONS •Better integration into the patient care pathway •Improved collaboration with oncology services and primary care •Improved collaboration with APs •Greater awareness of their service offering	FOR FACILITIES •Development of new patient follow-up methods •Fewer patients followed in facilities •Better use of hospital resources •Improved network integration •Development of expertise in involving APs at the clinical level	FOR THE MINISTRY Showcase for the “life after cancer” project •Research results to determine whether the model meets the needs and delivers the expected results •Fosters the network among all Quebec institutions	FOR SANTÉ QUÉBEC •Testing of a model to improve the transition from hospital to primary care •Participation in scaling up the model	FOR INESSS •Development of new reflective workshops on cancerology
Impacts	**On patient health** Improved mortality and morbidity Improved patient experience Improved quality of life Improved professional-patient relations		**On the well-being of professionals and managers** Improved practices Improved patient relations Improved work climate			**On cost reduction** Reduced consumption of care by patients (consultations, hospitalizations, medications, biological tests) Lower out-of-pocket costs for patients, with less travel and uncovered expenses Reduced costs associated with cancer follow-up		**On equity and inclusion** Better consideration for the differences among populations affected by breast cancer More diverse AP profiles			**On population health status** Better understanding of the post-treatment phase and the importance of social support Improved ability to return to the workforce Improved social capital	
